# Impaired speech perception in noise with a normal audiogram: No evidence for cochlear synaptopathy and no relation to lifetime noise exposure

**DOI:** 10.1016/j.heares.2018.03.008

**Published:** 2018-07

**Authors:** Hannah Guest, Kevin J. Munro, Garreth Prendergast, Rebecca E. Millman, Christopher J. Plack

**Affiliations:** a Manchester Centre for Audiology and Deafness, University of Manchester, Manchester Academic Health Science Centre, UK; b NIHR Manchester Biomedical Research Centre, Central Manchester University Hospitals NHS Foundation Trust, Manchester Academic Health Science Centre, UK; c Department of Psychology, Lancaster University, UK

**Keywords:** Speech in noise, Obscure auditory dysfunction, Cochlear synaptopathy, Hidden hearing loss, Auditory brainstem response, Envelope-following response, ABR, auditory brainstem response, AN, auditory nerve, AP, action potential, CRM, Coordinate Response Measure, EFR, envelope-following response, EHF, extended high frequency, NESI, Noise Exposure Structured Interview, SEM, standard error of the mean, SNR, signal-to-noise ratio, SPiN, speech perception in noise, SP, summating potential, SR, spontaneous rate, TTS, temporary threshold shift

## Abstract

In rodents, noise exposure can destroy synapses between inner hair cells and auditory nerve fibers (“cochlear synaptopathy”) without causing hair cell loss. Noise-induced cochlear synaptopathy usually leaves cochlear thresholds unaltered, but is associated with long-term reductions in auditory brainstem response (ABR) amplitudes at medium-to-high sound levels. This pathophysiology has been suggested to degrade speech perception in noise (SPiN), perhaps explaining why SPiN ability varies so widely among audiometrically normal humans. The present study is the first to test for evidence of cochlear synaptopathy in humans with significant SPiN impairment. Individuals were recruited on the basis of self-reported SPiN difficulties and normal pure tone audiometric thresholds. Performance on a listening task identified a subset with “verified” SPiN impairment. This group was matched with controls on the basis of age, sex, and audiometric thresholds up to 14 kHz. ABRs and envelope-following responses (EFRs) were recorded at high stimulus levels, yielding both raw amplitude measures and within-subject difference measures. Past exposure to high sound levels was assessed by detailed structured interview. Impaired SPiN was not associated with greater lifetime noise exposure, nor with any electrophysiological measure. It is conceivable that retrospective self-report cannot reliably capture noise exposure, and that ABRs and EFRs offer limited sensitivity to synaptopathy in humans. Nevertheless, the results do not support the notion that noise-induced synaptopathy is a significant etiology of SPiN impairment with normal audiometric thresholds. It may be that synaptopathy alone does not have significant perceptual consequences, or is not widespread in humans with normal audiograms.

## Highlights

• Study of adults with impaired speech perception in noise (SPiN) and normal audiograms.
• A subset of those with reported SPiN impairment exhibited measurable SPiN deficits.
• SPiN-impaired participants were matched with controls for age, sex, and audiogram.
• Impaired SPiN was not associated with ABR or EFR measures of cochlear synaptopathy.
• Impaired SPiN was not associated with a detailed measure of lifetime noise exposure.

## 1 Introduction

Some individuals exhibit pure tone audiometric thresholds within the clinically normal range, yet report deficits of speech perception in noise (SPiN). This profile describes a small but significant proportion of patients attending audiology services; amongst patients referred for hearing difficulties, subsequent findings of normal hearing thresholds have been reported in 5–8.4% (Saunders, 1989; Stephens et al., 2003). This presentation has been designated variously as “selective dysacusis” (Narula and Mason, 1988), “obscure auditory dysfunction” (Saunders and Haggard, 1989), “King-Kopetzky syndrome” (Hinchcliffe, 1992), “auditory disability with normal hearing” (King and Stephens, 1992), “idiopathic discriminatory dysfunction” (Rappaport et al., 1993), and “auditory processing disorder” (British Society of Audiology APD Special Interest Group, 2011). The present text will eschew these labels in favour of a descriptive term, “SPiN impairment with a normal audiogram”.

The relatively high prevalence of this clinical presentation has prompted a significant body of research into the underlying causes. Large-scale studies have revealed a heterogeneous condition, most probably with major contributions from psychological factors, alongside (or in combination with) auditory deficits (Saunders and Haggard, 1992; Zhao and Stephens, 2000). Even in those patients with genuinely impaired SPiN, there are many possible etiologies, including minor pathology of the middle ear or cochlea, impaired central auditory processing, and deficits of attention, memory, and/or language (for a review, see Pienkowski, 2017).

It is possible that new insight into SPiN impairment with a normal audiogram may be offered by the recent emergence of a pathophysiology termed “cochlear synaptopathy”: loss of synapses between inner hair cells and auditory nerve (AN) fibers, which can occur without widespread hair cell loss or permanent threshold elevation. Originally induced in mice by exposure to high-level noise (Kujawa and Liberman, 2009), synaptopathy has since been observed in noise-exposed guinea pigs, rats, and macaques, and in aging mice without purposeful noise exposure (for a summary of histological evidence, see Hickox et al., 2017). The synaptic damage appears to preferentially affect AN fibers with low-to-medium spontaneous rates (low-SR fibers; Furman et al., 2013), which have high response thresholds (Liberman, 1978). Cochlear thresholds are not permanently altered by the condition, though some loss of sensitivity at the highest frequencies can occur due to accompanying hair cell loss at the extreme cochlear base (Hickox et al., 2017). However, synaptopathy is associated with significant reductions in the amplitude of the auditory brainstem response (ABR) at moderate-to-high sound levels (Kujawa and Liberman, 2009).

It has been suggested that the suprathreshold effects of synaptopathy might also extend to auditory perception (Bharadwaj et al., 2014; Kujawa and Liberman, 2015; Plack et al., 2014). Kujawa and Liberman hypothesized that loss of low-SR fibers might largely explain why audiometrically normal individuals differ so widely in their SPiN abilities. The authors reasoned that, as background noise levels increase, humans must rely increasingly on these fibers, due to their large dynamic ranges and reduced susceptibility to noise masking. Accordingly, Lobarinas et al. (2017) have reported evidence consistent with perceptual effects of synaptopathy in rats. Noise exposures causing large temporary threshold shifts (TTS) led to post-TTS impairment of signal detection in noise and reduced ABR wave I amplitude. Deficits were limited to specific frequencies and low signal-to-noise ratios (SNRs) and were not well predicted by ABR effects, reducing confidence that the two were directly related. Nevertheless, the results provide the first experimental indication that noise exposure can alter hearing in noise while leaving threshold sensitivity intact.

Research in humans has yielded some evidence consistent with the existence of perceptually consequential synaptopathy. As will be outlined below, a number of studies have associated SPiN with noise exposure, with electrophysiological measures assumed to be sensitive to synaptopathy, or with both factors. However, other studies have revealed no such association. Moreover, some of the reported relations are not clearly reflective of underlying AN deficits and may be consistent with other pathologies.

Considering first the evidence in relation to noise exposure, several studies have reported poorer SPiN performance in occupationally noise-exposed individuals than in controls, though with possible contributions from uncontrolled audiometric hearing loss. Alvord (1983) reported that noise-exposure was associated with poorer discrimination of high-frequency monosyllables, but also with substantially poorer mean pure-tone thresholds (by 9.5 dB at 4 kHz). In the sentence recognition data of Kumar et al. (2012), audiometric thresholds merely fell in the range −10 to 25 dB HL and were neither matched between groups nor reported. In Hope et al. (2013), thresholds at individual frequencies were not reported or analyzed, nor measured beyond 4 kHz, and the apparent association between noise exposure and syllable recognition would not survive correction for multiple comparisons.

More recently, Yeend et al. (2017) investigated the effects of lifetime noise exposure on auditory processing in a large cohort (n = 122) with normal or near-normal audiometric thresholds. The survey of noise exposure incorporated both occupational and leisure noise exposure during each decade of life, with consideration given to duration and level of exposure and to the effects of hearing protection. Participants also completed several measures of temporal and spectral processing and two SPiN tasks. No relation of noise exposure to any perceptual measure was evident.

Perhaps most relevant to the present research is the large-scale clinical study reported by Stephens et al. (2003), examining self-reported noise exposure in patients with King-Kopetzky syndrome (that is, SPiN impairment with a normal audiogram). The study recruited a very large SPiN-impaired cohort (n = 110), though “normal hearing” was defined less strictly than in most synaptopathy research (≤20 dB HL at 0.5–4 kHz and ≤30 dB HL at 0.25–8 kHz). Controls (n = 70) met the same audiometric criteria and had similar age and sex distributions. Participants completed an etiological-factors questionnaire with a principal focus on noise exposure history. SPiN impairment was not associated with noise exposure.

Other researchers have sought to relate SPiN primarily to electrophysiological measures of synaptopathy. Bharadwaj et al. (2015) demonstrated correlations between the subcortical envelope-following response (EFR) and behavioral measures of temporal coding, including a spatial digit-discrimination task reliant on temporal cues. Bharadwaj and colleagues recorded EFRs to various modulation depths, allowing computation of a difference measure designed to emphasize the contributions of low-SR fibers. The resulting correlations suggest that perceptual abilities are partially determined by individual differences in temporal coding fidelity early in the neural pathway. Cochlear synaptopathy was suggested as a possible mechanism underlying this variability, an interpretation bolstered by marginal associations with a rudimentary measure of noise exposure.

Bramhall et al. (2015) analyzed relations between ABR wave I amplitude and sentence perception in noise in a large cohort of listeners with a broad array of audiometric profiles. A substantial subset exhibited normal or near-normal hearing sensitivity. ABR amplitude was correlated with SPiN, but the correlation was driven by audiometric differences; linear mixed-effects modelling revealed no main effect of ABR amplitude on performance, either in the full group or in the subset with acute hearing sensitivity.

Finally, several recent studies have combined measures of noise exposure, SPiN, and brainstem-response amplitudes with the explicit aim of investigating noise-induced synaptopathy. The first, conducted by Liberman et al. (2016), divided 34 students into high- and low-risk groups based on a short questionnaire assessing noise exposure habits. The high-risk group exhibited poorer word recognition in noise, along with elevated values of an electrocochleographic measure: the ratio of summating potential amplitude to action potential amplitude (SP/AP ratio). Results were interpreted as evidence of noise-induced synaptopathy with effects on SPiN. However, the high-risk group exhibited a substantial deficit in extended-high-frequency (EHF) audiometric sensitivity relative to the low-risk group (∼20 dB at 16 kHz). Basal dysfunction may have influenced the electrocochleographic results, since stimuli were presented at an extremely high level, 130 dB peSPL. Consistent with this interpretation, the resulting enhancement of SP/AP ratio in the high-risk group was driven largely by higher SP (a primarily pre-neural potential), with no significant reduction evident in AP (reflective of AN activity). Speech stimuli were delivered at 35 dB HL, a sound level insufficient to emphasize the contributions of high-threshold fibers and perhaps more sensitive to other pathologies.

Prendergast et al. (2017b) used a detailed structured interview to quantify lifetime noise exposure in 141 audiometrically normal young listeners. Participants completed SPiN tasks which varied in sound level and reliance on spatial cues, allowing computation of within-subject difference measures designed to enhance sensitivity to synaptopathy (Plack et al., 2016). Additional psychoacoustic tasks included frequency and intensity difference limens, inter-aural phase difference discrimination, and amplitude modulation detection. After correction for multiple comparisons, noise exposure exhibited no significant relation with any behavioral measure. ABR and EFR measures in a near-identical cohort were previously reported (Prendergast et al., 2017a) and were not associated with noise exposure.

Grose et al. (2017) administered a similarly extensive test battery in two groups of audiometrically normal young people, differing greatly in their exposure to loud music events. High-noise participants (n = 31) had attended a median of 90 such events in the preceding two years, while low-noise participants (n = 30) had attended four. The high-noise group demonstrated a reduction in ABR wave I/V amplitude ratio, though this would not survive correction for multiple comparisons and was accompanied by an EHF audiometric deficit (∼10 dB at 16 kHz), whose effects on the ABR are unknown. Noise exposure was not significantly associated with wave I amplitude, EFR amplitude, EFR slope, or with performance on any listening task, including sentence recognition in noise. The authors concluded that, even if noise-induced synaptopathy is manifest in humans, its perceptual consequences may be so insignificant as to elude detection.

Grinn et al. (2017) investigated effects of recreational noise exposure both retrospectively and prospectively, assessing auditory function before and after a loud music event and also obtaining self-report of noise exposure over the past 12 months. AP amplitude, SP/AP ratio and SPiN were not associated with previous-12-months’ noise exposure, nor reduced following a single exposure. However, it is not clear that a single exposure would be expected to cause measurable synaptopathy, given that participants had experienced many such exposures. Additionally, statistical power in the retrospective analysis was limited by the small sample (n = 32).

Fulbright et al. (2017) also recorded previous-12-months’ noise exposure, this time from 60 young, normally hearing participants. Participants also underwent tests of word recognition (in broadband noise and in multitalker babble) and ABRs at 70, 80, 90, and 99 dB nHL. ABR wave I amplitude was not significantly related to noise exposure, nor to perceptual performance.

Taken together, evidence for noise-induced synaptopathy as a determinant of speech perception appears tenuous. One possible explanation is that researchers have not purposely recruited individuals with significant deficits of speech perception, leading to cohorts with relatively homogeneous perceptual performance. Investigation of synaptopathy in individuals with SPiN impairment therefore represents an important gap in the literature. Careful control of audiometric thresholds should also be a priority, since audiometric influences on both electrophysiological and perceptual measures are possible. Interpretation of much existing synaptopathy research is complicated by this potential confound (Guest et al., 2017b).

The present study aimed to test for associations between SPiN impairment with a normal audiogram and (a) ABR measures of cochlear synaptopathy, (b) EFR measures of synaptopathy, and (c) lifetime noise exposure. We reasoned that such associations would together constitute plausible non-invasive evidence for noise-induced cochlear synaptopathy, if audiometric, sex, and age differences between groups were minimized. To enhance the likelihood of observing such evidence, the research questions were addressed primarily in a cohort with “verified SPiN impairment”: that is, presenting with both self-reported and laboratory-measured SPiN deficits.

## 2 Material and methods

### 2.1 Participants

Control participants were recruited from the University of Manchester staff and student population (via poster and on-line advertising) and from the general population of Greater Manchester (via on-line advertising). Participants with SPiN impairment were recruited from local audiology services and from the sources above. All were aged 18–40 and were fluent English speakers, either monolingual or early bilingual (acquired both languages by age 12 years). All exhibited normal otoscopic findings, normal pure-tone audiometric thresholds (≤20 dB HL at 0.25–8 kHz), and reported no history of middle-ear surgery, neurological disorder, head trauma, or ototoxic exposure. For all but two participants, tympanometric results were within clinically normal limits (compliance 0.3–1.6 cm^3^, pressure −50 to +50 daPa). The exceptions were one control participant (2.4 cm^3^ compliance unilaterally) and one participant with SPiN impairment (0.2 cm^3^ compliance bilaterally). In both cases, bone conduction audiometry revealed no significant air-bone gaps (≤5 dB at all but two test frequencies, and ≤10 dB at all test frequencies) and acoustic reflex testing at 1 and 2 kHz yielded thresholds <95 dB HL bilaterally.

Potential recruits to the SPiN-impairment group (n = 47) were recruited based on self-report of significant difficulties understanding speech in complex auditory environments (more than their peers) and subsequently provided a brief history of the nature and time course of their hearing deficits (summarized in supplementary material, Table SM1). Fifteen were excluded at the screening stage on the basis of audiological history, middle ear function, and/or pure-tone audiometry. The remaining 32 comprised the reported-SPiN-impairment group. Of these, 16 progressed to a verified-SPiN-impairment group, based on a laboratory SPiN measure (see Section 2.2.2). Eleven participants with reported SPiN impairment and six participants with verified SPiN impairment also reported tinnitus. Potential control participants (n = 38) reported no self-perceived auditory deficits (significant listening difficulties or tinnitus). Controls drawn from this initial group were matched with SPiN-impaired participants on the basis of age, sex, and audiometric thresholds (Section 2.6 provides information on matching).

In the study's main analysis (see Table 1), participants with verified SPiN impairment were compared with controls matched for audiometric thresholds up to 14 kHz. The decision to focus on participants with *verified* SPiN impairment was motivated by evidence that some individuals with reported SPiN impairment underestimate their hearing ability (Saunders and Haggard, 1992). The decision to match audiograms to 14 kHz was motivated by concerns over a possible confound, since loss of basal sensitivity might be associated with poorer perceptual performance (Yeend et al., 2017) and affect electrophysiological responses (Don and Eggermont, 1978; Hardy et al., 2017). Section 2.6 describes two supplementary analyses, which address parallel research questions using (a) the cohort with “reported SPiN impairment” (n = 32), and (b) non-audiogram-matched controls.

**Table 1:** Participant characteristics.

Analysis	Participant group	n	Female	Mean age (years)	Mean 14 kHz audiometric threshold (dB SPL)	Median CRM threshold (dB)	Results reported in …
*Main analysis*	Verified SPiN impairment	16	10 (63%)	27.6	48.0	−10.5	Main paper
	Closely audiogram-matched controls	16	10 (63%)	28.4	45.9	−16.0	
*Supplementary analysis 1*	Reported SPiN impairment	32	18 (56%)	26.6	44.2	−13.9	Supplementary material
	Closely audiogram-matched controls	32	18 (56%)	27.8	41.4	−16.9	
*Supplementary analysis 2*	Verified SPiN impairment	16	10 (63%)	27.6	48.0	−10.5	Supplementary material
	Non-audiogram-matched controls	16	10 (63%)	27.6	42.4	−16.7	

### 2.2 Perceptual measures

#### 2.2.1 Audiometry

Methods were as reported in Guest et al. (2017a). Pure-tone air-conduction thresholds at 0.25–8 kHz were obtained in accordance with British Society of Audiology (2011) recommended procedures. EHF thresholds at 10 and 14 kHz were obtained using 1/3-octave noise bands, in order to limit the influence of ear canal resonances and threshold microstructure (periodic fluctuations in threshold with small changes in signal frequency). At both standard and extended high frequencies, thresholds were obtained for each ear separately, then averaged between ears.

#### 2.2.2 Speech perception in noise: the coordinate response measure (CRM)

We aimed to design a SPiN measure that (a) possessed key attributes of the challenging listening situations reported by individuals with impaired SPiN and normal audiograms, and (b) emphasized the auditory structures and processes thought to be impaired by cochlear synaptopathy. In pursuit of the first aim, the measure incorporated meaningful speech stimuli (as opposed to nonsense syllables), high overall sound levels, competing talkers, and spatial cues. The latter three attributes were also expected to enhance sensitivity to synaptopathy, since loss of low-SR fibers should degrade the subtle temporal and level cues required to encode spatial information, especially at high sound levels. To enhance the specificity of the measure to auditory deficits, we selected a closed-set task incorporating simple vocabulary, in common with Bharadwaj et al. (2015). This was intended to reduce the influence of linguistic factors, rendering the measure appropriate for use in multilingual populations and relatively insensitive to SPiN deficits arising from language disorders.

Speech stimuli and speech maskers were CRM phrases, of the form “Ready {call-sign}, go to {color} {number} now”, spoken by native British-English talkers (Kitterick et al., 2010). Each trial included one target phrase with call-sign “Baron”, concurrent with two masker phrases containing other call-signs. The phrases (sampled at 44.1 kHz) were spatialized through convolution with head-related impulse responses from the CIPIC database (Algazi et al., 2001) prior to presentation through Sennheiser HD650 circumaural headphones, driven by an E-MU 0202 audio interface. Target, Masker 1, and Masker 2 were presented at 0°, −60°, and +60° azimuth, respectively.

Participants were instructed to report the color (red, white, green, or blue) and number (1, 2, 3, or 4) spoken by the target talker: a one-interval, 16-alternative, forced-choice procedure. Responses were made via a mouse and visual display and feedback was provided after each response. Talker identity for target and masker phrases varied between trials, drawn randomly from eight talkers (four male), with the constraint that no trial could contain more than one instance of a given talker. Combined masker level remained constant at 80 dB SPL, while target level varied adaptively. A one-down, one-up decision rule targeted 50% correct performance, over the course of four initial turnpoints (4 dB step size) and eight subsequent turnpoints (2 dB step size). The SNR at the final eight turnpoints was averaged to yield threshold. Two such adaptive tracks were measured for each participant and the resulting thresholds averaged. Prior to threshold measurement, participants completed two practice tracks, each containing eight turnpoints. Participants with reported SPiN impairment were included in the verified-SPiN-impairment group if their CRM thresholds fell at or above the 90^th^ percentile of control thresholds.

### 2.3 Educational level and cognitive ability

Since cognitive factors may contribute to SPiN deficits (Pienkowski, 2017), brief assessments of educational attainment and cognitive function were conducted. Participants reported the highest educational level at which they had studied and whether or not they had completed the course of study in question. Based on this report, they were assigned to one of the following ordinal categories: doctoral graduate, doctoral student, master's graduate, master's student, bachelor's graduate, bachelor's student, or no higher education. Participants also completed both parts of the neuropsychological Trail Making Test, using pen and paper and following the protocol of Bowie and Harvey (2006). Participants drew lines to connect pseudo-randomly distributed numerals and letters in a specified order, proceeding as rapidly and accurately as possible. The first part, in which numerals are connected in ascending order, is thought to assess psycho-motor speed and visual search skills. The second, which alternates between numerals and letters (1-A-2-B-3-C, *etc*.), is thought to additionally assess higher level cognitive skills such as mental flexibility, though correspondence of performance to any discrete cognitive domain is uncertain (Crowe, 1998). Prior to testing, participants completed short practice versions of each part.

### 2.4 Lifetime noise exposure: the noise exposure structured interview (NESI)

Methods were as reported in Guest et al. (2017a). In summary, the NESI directs respondents to (i) identify occupational and/or recreational noisy activities (>80 dBA) in which they have engaged; (ii) for each activity, identify life periods in which exposure habits have been approximately stable; (iii) estimate exposure duration for each period, based on frequency of occurrence and duration of a typical exposure; (iv) estimate exposure level, based on vocal effort required to hold a conversation or, for personal listening devices, typical volume control setting; (v) report usage and type of hearing protective equipment. The resulting data from all activities and life periods are combined to yield units of lifetime noise exposure, a measure linearly related to the total energy of exposure above 80 dBA. Further details are provided in the supplementary material (Table SM2 lists the conversion values used in estimating sound level; Table SM3 provides the NESI calculation for a single participant).

### 2.5 Electrophysiological measures

Methods were largely as reported in Guest et al. (2017a) and are stated in full on page 6 of the supplementary material, with key elements summarized below.

#### 2.5.1 Auditory brainstem response

Stimuli were filtered clicks designed to focus excitation on the characteristic frequencies typically affected by early noise-induced cochlear damage. The stimuli had a 10 dB bandwidth extending from 1.2 to 4.7 kHz (as recorded in a Gras IEC60711 occluded-ear simulator) and were delivered at 102 dB peSPL, sufficient to elicit the half-octave basalward shift in the travelling wave (McFadden, 1986) and provide strong excitation of characteristic frequencies between approximately 2 and 7 kHz. Each ear received 7040 stimuli at a rate of 7.05/second. Recording montage was Cz to ipsilateral mastoid and responses were band-pass filtered between 50 and 1500 Hz. Waves I and V of the averaged waveform were identified by a peak-picking algorithm (wave I falling at 1.55–2.05 ms after stimulus peak, wave V at 5.1–6.6 ms). Post-hoc subjective review verified that the algorithm had appropriately interpreted all waveforms (presented in full on pages 7 and 8 of the supplementary material). For all participants but one, the amplitudes of wave I (peak-trough) and V (peak-baseline) were obtained for both ears, then averaged between ears. For one participant (a member of the reported-SPiN-impairment group but not the verified-SPiN-impairment group), only the left ABR was analyzed, due to a technical fault during recording.

#### 2.5.2 Envelope-following response

**Fig. 1:**
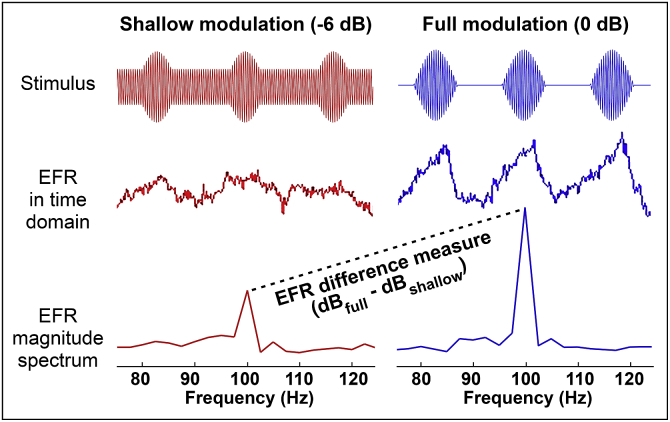
A schematic illustration of the EFR paradigm, including responses and response spectra from a single participant. Raw response amplitude at 100 Hz was analyzed, along with an EFR difference measure comparing response amplitudes at two stimulus modulation depths. It was predicted that loss of low-SR fibres should primarily impair responses at the shallow modulation depth, leading to higher values of the difference measure in synaptopathic ears.

Stimuli were transposed tones (Bernstein and Trahiotis, 2002) with the same carrier frequency, modulation frequency, off-frequency masking characteristics, presentation level, stimulus duration, and ramp duration as used by Bharadwaj et al. (2015). Inter-stimulus interval was 400 ms and the recording channel was Cz to C7. The tones were of two modulation depths: 0 dB (full modulation) and −6 dB (shallow modulation). This approach allowed computation of an EFR difference measure: the difference in response amplitude (in dB) at the two stimulus modulation depths. This measure is closely related to the “EFR slope” metric of Bharadwaj and colleagues, though based on a two-point function, and reflects the assumption that synaptopathy preferentially affects high-threshold AN fibers and should therefore preferentially degrade the encoding of stimuli with shallow modulations. A schematic illustration of the difference measure is provided in Fig. 1. Since it is possible that responses to both modulation depths might be impaired by synaptopathy, raw response amplitude was also analyzed.

### 2.6 Analysis

The main analysis compared participants with verified SPiN impairment (n = 16) with controls (n = 16) matched on the basis of age, sex, and audiometric thresholds up to 14 kHz. Controls (n = 4) with poor SPiN performance (CRM thresholds >90^th^ percentile) were excluded from the reservoir of potential matches. Matching aimed to minimize the difference in mean 14 kHz thresholds between the groups while allowing mean age to differ by no more than 1 year. Characteristics of the resulting groups are reported in Table 1. Each research question was addressed in R (R Core Team, 2015) by way of independent-samples Student's *t*-test, unequal variance *t*-test, or Wilcoxon-Mann-Whitney test, as appropriate. All significance tests were two-tailed. The exception was the EFR analysis, which employed a mixed two-way ANOVA with group as the between-subjects variable and stimulus modulation depth as the within-subject variable.

Two supplementary analyses were performed. The first compared participants with reported SPiN impairment (n = 32) with age-, sex-, and audiogram-matched controls (n = 32). This approach allowed our research questions to be addressed in a SPiN-impaired sample defined by self-report, which is arguably more relevant to clinical presentations of SPiN impairment than a sample defined by lab-measured performance. The second was a comparison of the verified-SPiN-impairment group with controls matched only for age and sex, not for audiometric thresholds (controls were selected to provide optimal age-matching, allowing thresholds to vary freely.) This approach was informed by the suggestion that high-frequency audiometric loss might be a biomarker for cochlear synaptopathy at lower frequencies (Liberman et al., 2016), meaning that audiometric over-matching might obscure relations between SPiN impairment and synaptopathy. Core outcomes of these supplementary analyses are reported in the main text, while figures and further statistics are reported on pages 1 and 2 of the supplementary material.

## 3 Results

### 3.1 Audiometry

**Fig. 2:**
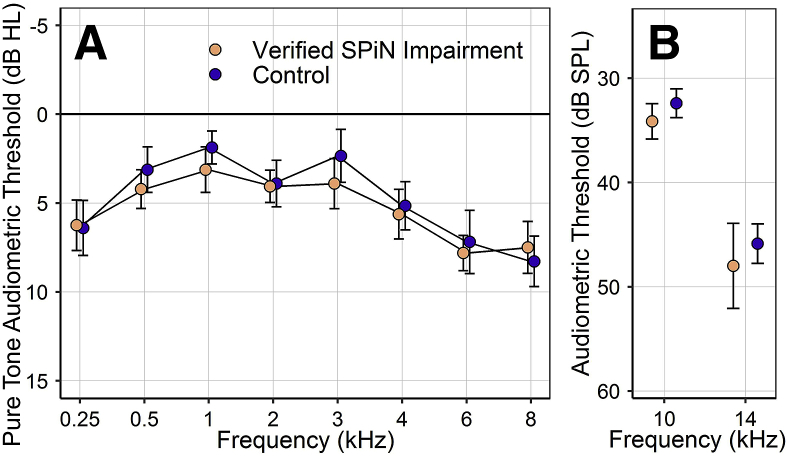
Mean audiometric thresholds for the verified-SPiN-impairment and control groups. Error bars represent the standard error of the mean (SEM). **A:** Pure-tone audiometric thresholds. Group means differ by < 2 dB. **B:** EHF audiometric thresholds for 1/3-octave narrowband noise. Group means differ by 1.7 dB at 10 kHz and 2.1 dB at 14 kHz.

For the groups used in the main analysis, audiometric thresholds were closely matched. The difference in mean threshold between verified-SPiN-impairment and control groups was <2 dB for pure tones at 0.25–8 kHz (Fig. 2A) and <2.2 dB for EHF thresholds at 10 and 14 kHz (Fig. 2B). Similar results were obtained in the first supplementary analysis, comparing the reported-SPiN-impairment group with controls. For the final supplementary analysis, participants with verified SPiN impairment and controls were not purposely audiogram-matched, yielding groups whose mean thresholds differed by 3.1 dB at 8 kHz, 4.2 dB at 10 kHz, and 5.6 dB 14 kHz, but differed little at lower frequencies (see page 2 of the supplementary material for audiograms).

### 3.2 Speech perception in noise

**Fig. 3:**
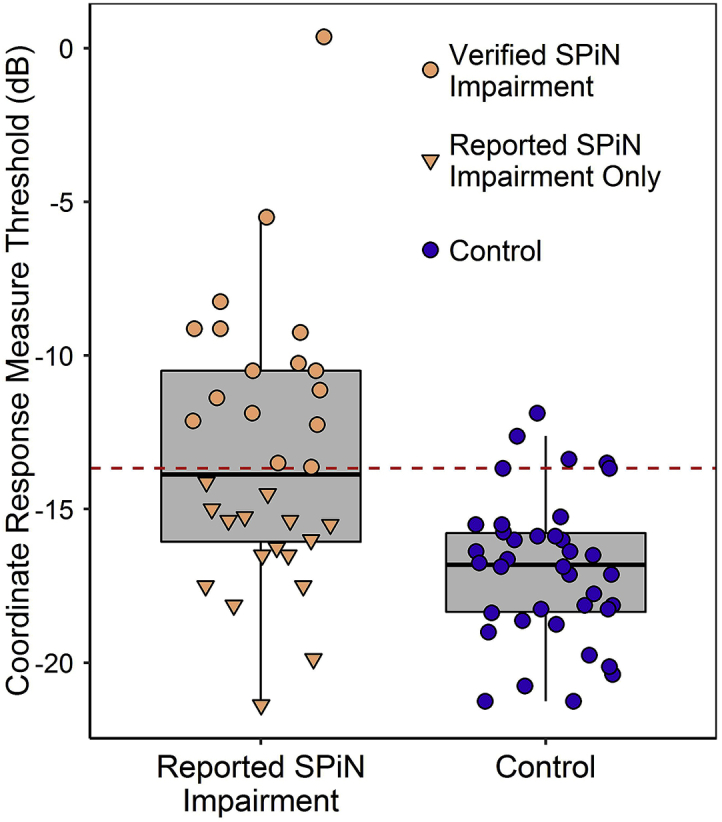
Thresholds recorded for the CRM: a measure of SPiN involving high sound levels, multiple talkers, and spatial cues. Points correspond to individual participants, upper and lower hinges to first and third quartiles, upper whiskers to the highest value within 1.5 * IQR of the upper hinge (where IQR is the interquartile range), and lower whiskers to the lowest value within 1.5 * IQR of the lower hinge. The horizontal dashed line represents the criterion for inclusion in the verified-SPiN-impairment group: thresholds at or above the 90^th^ percentile of control thresholds.

SPiN performance among participants with reported SPiN impairment exhibited substantial inter-subject variability (Fig. 3). CRM thresholds ranged from −21.4 dB (surpassing even the best-performing control) to 0.4 dB (a deficit of 17 dB relative to median control threshold). Only half of the participants with reported SPiN impairment (n = 16) met the criterion for inclusion in the verified-SPiN-impairment group, consistent with past reports of underestimation of hearing ability in this population (Saunders and Haggard, 1992).

### 3.3 Educational level and cognitive ability

Verified-SPiN-impairment and control groups were similarly educationally diverse, with no indication of higher educational status among the control participants. Analysis by Wilcoxon-Mann-Whitney test indicated no significant between-groups differences in the distributions of participants among the educational categories (*U* = 105, *p* = 0.37). The time taken to complete Part B of the Trail Making Test did not differ significantly between groups (*t*(30) = −0.71, *p* = 0.49), providing no indication of cognitive contributions to SPiN impairment. The same patterns of educational and cognitive results were obtained in both supplementary analyses (see pages 1 and 2 of the supplementary material).

### 3.4 Lifetime noise exposure

**Fig. 4:**
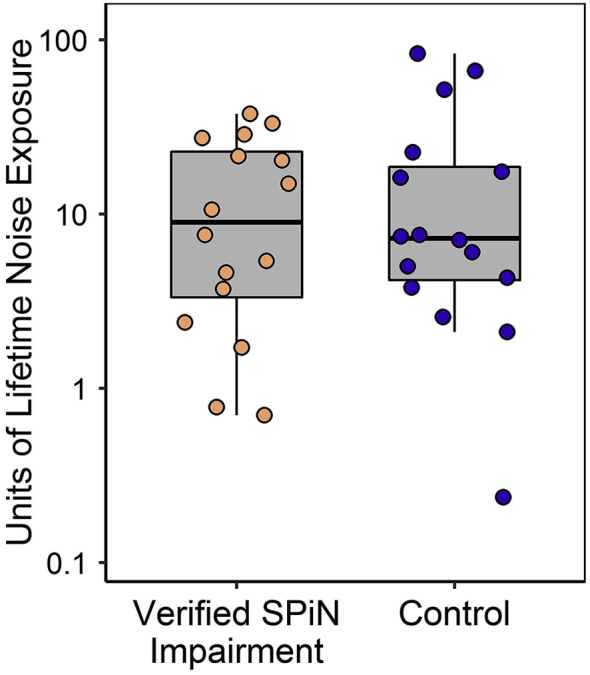
NESI units of lifetime noise exposure (linearly related to total energy of exposure >80 dBA) for verified-SPiN-impairment and control groups. Points correspond to individual participants, upper and lower hinges to first and third quartiles, upper whiskers to the highest value within 1.5 * IQR of the upper hinge, and lower whiskers to the lowest value within 1.5 * IQR of the lower hinge.

Fig. 4 illustrates NESI units of lifetime noise exposure. Note that these units (presented here on a logarithmic scale) are linearly related to total energy of exposure and range from 0.1 to 90, indicating a wide range of exposures in this cohort (a factor of 900 in energy between the lowest and highest exposed). Noise exposure did not differ significantly between participants with verified SPiN impairment and controls (*U* = 125, *p* = 0.93, Wilcoxon-Mann-Whitney test), a finding repeated in both supplementary analyses (see pages 1 and 2 of the supplementary material).

### 3.5 Auditory brainstem response

**Fig. 5:**
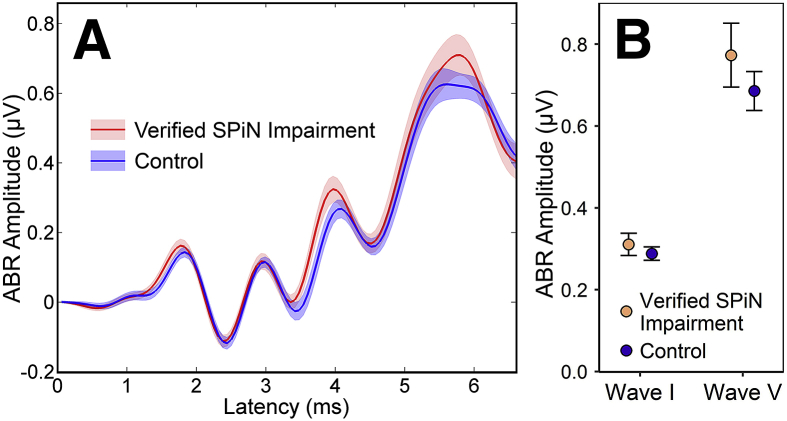
ABRs elicited by 102 dB peSPL clicks for verified-SPiN-impairment and control groups. **A:** Grand average waveforms (averaged across ears and across participants). Shaded areas represent the SEM. **B:** Wave I and wave V amplitudes, presented as mean ± SEM.

Fig. 5 illustrates the ABR data obtained from participants with verified SPiN impairment and closely audiogram-matched controls. ABR wave I amplitude did not differ significantly between the groups (*t*(30) = 0.7, *p* = 0.49). A second ABR measure was also computed: the ratio of wave I amplitude to wave V amplitude, which has been suggested as a self-normalized measure of AN function with potentially enhanced sensitivity to synaptopathy (Schaette and McAlpine, 2011). No association with verified SPiN difficulties was evident (*U* = 128, *p* = 0.99, Wilcoxon-Mann-Whitney test). Neither wave I amplitude nor the ratio measure differed between groups in either supplementary analysis (see pages 1 and 2 of the supplementary material).

### 3.6 Envelope-following response

**Fig. 6:**
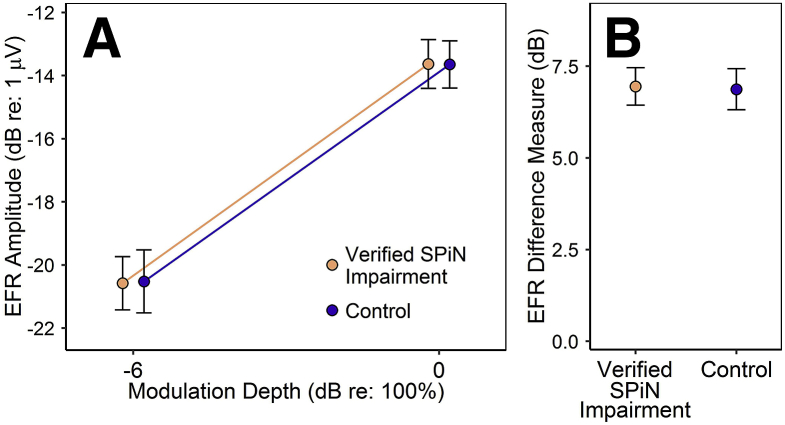
EFRs to stimuli of two modulation depths for verified-SPiN-impairment and control groups. **A:** EFR amplitudes (in dB re: 1 μV), presented as mean ± SEM. **B:** The difference in response amplitude at the two modulation depths (mean ± SEM).

Response SNR exceeded 6 dB for 100% of EFRs at the full stimulus modulation depth, and for 91.4% at the shallow modulation depth (90.6% of SPiN-impaired participants, 92.1% of controls). In the main analysis (and in both supplementary analyses), response amplitudes (expressed in dB re: 1 μV) were normally distributed at both modulation depths in both participant groups, and hence were analyzed by a mixed two-way ANOVA, with group as the between-subjects factor and stimulus modulation depth as the within-subject factor. The model revealed a highly significant effect of stimulus modulation depth (*F*(1,30) = 333, *p* < 0.001), but no significant effect of group (*F*(1,30) = 0.00, *p* = 0.99) and no significant interaction effect (*F*(1,30) = 0.01, *p* = 0.92). Hence, as can be seen from Fig. 6, verified SPiN impairment was not associated with reduced EFR amplitude, nor with rapid declines in amplitude with decreasing modulation depth. These results were echoed in both supplementary analyses (see pages 1 and 2 of the supplementary material).

## 4 Discussion

Humans with impaired SPiN and normal audiometric thresholds were matched with controls on the basis of age, sex, and audiometric sensitivity. In the main analysis, SPiN impairment was defined both by self-report and laboratory SPiN performance, and audiometric thresholds were matched closely up to 14 kHz. This design was chosen because some apparently SPiN-impaired patients underestimate their listening abilities (Saunders and Haggard, 1992) and because even minor high-frequency hearing loss may impact electrophysiological measures of synaptopathy (Verhulst et al., 2016; Hardy et al., 2017). In addition, one supplementary analysis defined SPiN impairment solely by self-report and a second supplementary analysis allowed audiometric thresholds to differ between groups. SPiN impairment was not associated with lifetime noise exposure, nor with any ABR or EFR measure of synaptopathy, despite small standard errors. These findings were consistent across all three analyses.

Such uniformly null results appear at odds with the notion that noise-induced cochlear synaptopathy is a significant etiology of impaired SPiN with a normal audiogram. The present study is, to the authors’ knowledge, the first to investigate synaptopathy in individuals with significant listening difficulties. However, its results accord with an existing body of literature that finds little evidence for relations of SPiN to noise exposure and AN function, or finds evidence that could reasonably be attributed to pathologies other than synaptopathy. Links between brainstem response measures and perceptual performance have been reported by some (Bharadwaj et al., 2015; Liberman et al., 2016), but not others (Bramhall et al., 2015; Fulbright et al., 2017). Reported relations of SPiN to occupational noise exposure are complicated by the possible influence of audiometric deficits (Alvord, 1983; Kumar et al., 2012; Hope et al., 2013). Two small studies of college students found no relation of noise exposure to SPiN (Le Prell and Lobarinas, 2016; Grinn et al., 2017). In a third, noise exposure was associated with poorer SPiN, but at a low sound level unlikely to emphasize low-SR fibers (Liberman et al., 2016). A clinical study of SPiN impairment in 110 patients with normal audiograms demonstrated no relation to noise exposure history (Stephens et al., 2003). Finally, large-scale studies aiming to investigate noise-induced synaptopathy have revealed no effects of noise exposure on a broad array of perceptual measures (Prendergast et al., 2017b; Grose et al., 2017; Fulbright et al., 2017; Yeend et al., 2017).

The dearth of consistent evidence for perceptually consequential synaptopathy in humans is surprising, given histological evidence for the pathophysiology in animal models. Possible explanations for the present results must be considered carefully. Chief among them are: (a) cochlear synaptopathy is not widespread in young people with normal audiometric thresholds; (b) cochlear synaptopathy does not substantially degrade SPiN; (c) our measures of cochlear synaptopathy and noise exposure are not sufficiently sensitive.

### 4.1 Possibility A: cochlear synaptopathy is not widespread in young people with normal audiometric thresholds

In numerous rodent models, cochlear synaptopathy has been induced in young animals by exposure to high-level noise, without permanent elevation of cochlear thresholds. Translation of these findings to humans may not be straightforward. In animals, exposures are carefully titrated so as to maximize synaptopathy without widespread hair-cell loss; even so, some loss of sensitivity tends to result, albeit restricted to the extreme cochlear base (e.g. Kujawa and Liberman, 2009; Liberman et al., 2015; Shaheen et al., 2015). Since human exposures are far more diverse, synaptopathy without audiometric loss may be rare. Susceptibility to synaptopathy may also be far lower in humans than in rodents, since inter-species differences are apparent even among animal models. In comparison to in-bred mice, guinea-pigs incur synaptopathy at higher sound levels (Furman et al., 2013; Shi et al., 2013) and their synapses appear to regenerate in the weeks following exposure (Shi et al., 2013). In macaques, a high sound level of 108 dB SPL produced relatively modest synaptic loss (12–27% in basal regions), accompanied by mild outer hair cell loss (Valero et al., 2017). Based on analogous TTS studies in mice and humans, Dobie and Humes (2017) estimate that noise-induced synaptopathy in humans might require a 2-h exposure level of ∼114 dB SPL. In light of probable human resilience to synaptopathy, the findings of Maison et al. (2013) gain fresh significance, since they suggest that longer-duration exposures to moderate sound levels are also synaptopathic. However, it is not clear that synaptopathy was present in the latter study; synaptic densities of exposed animals were similar to those of control animals in previous studies (Le Prell and Brungart, 2016).

Evidence for noise-induced synaptopathy in audiometrically normal humans relies on non-invasive proxies, and remains inconclusive. An apparent negative relation between ABR wave I amplitude and previous-12-months’ noise exposure was sex-confounded (Stamper and Johnson, 2015a). Upon reanalysis, the relation remained only for females at the highest stimulus level; males exhibited an opposing trend (Stamper and Johnson, 2015b). Basal influences are unknown, since EHF audiometric thresholds were not measured. The high-noise participants of Liberman et al. (2016) did not demonstrate significantly reduced AP amplitude, and it is not clear that their enhanced SP/AP ratio is more consistent with synaptopathy than other forms of cochlear damage. Prendergast et al. (2017a) found no electrophysiological evidence for noise-induced synaptopathy in a cohort of 126, using both ABR and EFR measures. The Bayesian regression analysis of Bramhall et al. (2017) associated noise exposure with wave I amplitude, but it is not clear that audiometric and sex confounds were adequately controlled. An informative prior was not specified for the expected effects of sex on amplitude, despite a pronounced correlation between sex and noise-exposure group. Audiometric thresholds were omitted from the model entirely, despite a 7.3 dB disparity between the highest- and lowest-exposed groups. Guest et al. (2017a) found no association between lifetime noise exposure and ABR or EFR measures of synaptopathy. The high-noise group in the study by Grose et al. (2017) exhibited lower values of the ABR wave I/V amplitude ratio (*p* = 0.03, uncorrected), though not of ABR wave I amplitude, nor any EFR measure. In the data of Spankovich et al. (2017), noise history was not associated with ABR wave I amplitude, nor wave I/V amplitude ratio, measured using both high and low click rates. Grinn et al. (2017) observed no relation between ABR wave I amplitude and noise exposure, either reported for the previous 12 months or incurred at a single loud-music event, though the sample was small (n = 32). In Fulbright et al. (2017), previous-12-months’ noise exposure was not associated with ABR wave I amplitude at any of four stimulus levels. Whilst histology provides support for the existence of age-related synaptopathy in humans (Makary et al., 2011; Viana et al., 2015), evidence in relation to noise exposure is less convincing, reducing confidence that synaptopathy is prevalent in young, audiometrically normal humans.

### 4.2 Possibility B: cochlear synaptopathy alone does not substantially impair SPiN

Kujawa and Liberman (2015) hypothesized that synaptopathy might explain SPiN deficits in humans with normal audiograms, citing the likely importance of low-SR, high-threshold fibers for listening in background noise. However, this reasoning rests upon the assumptions that synaptopathy in humans preferentially affects low-SR fibers, and that low-SR fibers in humans possess high response thresholds. The latter assumption, in particular, may be unfounded. Hickox et al. (2017) note that the low-SR/high-threshold relation observed in the AN fibers of mice, gerbils, guinea-pigs, and cats may not hold true in primates. Single-unit recordings from the AN fibers of macaque monkeys have demonstrated no systematic relation between SR and threshold (Joris et al., 2011).

If synaptopathy in humans does not preferentially affect high-threshold fibers, then its impact on perception may be limited. Oxenham (2016) devised a simple model based on signal detection theory to predict the effects of mixed-SR synaptopathy on tone detection in quiet and in noise and on the discrimination of frequency, intensity, and inter-aural time differences. For all measures, a 50% loss of AN fibers was predicted to produce barely measurable effects on performance. On the other hand, Lopez-Poveda and Barrios (2013) have suggested that widespread synaptic loss might degrade SPiN regardless of fiber type, by leading to a “stochastically undersampled” neural representation of the sound waveform. However, the vocoder used to test this hypothesis may not have meaningfully simulated the effects of synaptopathy (Oxenham, 2016).

Finally, it is important to note that myriad factors besides cochlear function influence speech perception, including the function of the central auditory pathways, linguistic abilities, attention, and working memory (Pienkowski, 2017; Yeend et al., 2017). Even if cochlear synaptopathy has effects on SPiN, and especially if these effects are modest, it is conceivable that they might be eclipsed by variability in other factors.

### 4.3 Possibility C: our measures of cochlear synaptopathy and noise exposure are insufficiently sensitive

Of the dependent measures employed in the present study, the NESI appears most questionable, given the inherent inaccuracy and unreliability of retrospective self-report (Sallis and Saelens, 2000). However, cross-sectional investigations of noise-induced synaptopathy are bound to rely on such data, at least in societies where workplace regulations limit the contribution of occupational noise to the lifetime noise dose. A reasonable question, then, is how the design of the NESI compares to the alternatives, and especially to those measures successfully associated with putative measures of synaptopathy.

Bharadwaj et al. (2015) employed a rudimentary noise metric that was supplementary to the study's main measures, but whose methods were clearly reported. Participants rated their degree of exposure for four common noisy activities, along with their past experience of TTS. Scores were combined by weighting all categories equally. A much wider range of potentially noisy activities is surveyed by the Noise Exposure Questionnaire (Stamper and Johnson, 2015a; Johnson et al., 2017), which also considers frequency and duration of exposure and use of hearing protection. However, these data are obtained for only the past 12 months of exposure. Hence, the measure is unsuitable for assessing cumulative exposure, and is likely to be especially inappropriate for use with respondents whose exposure habits have changed markedly over the years. The brief questionnaire administered by Liberman et al. (2016) addressed both social and occupational noise exposure. For each, it sought information on number of years of exposure, proportion of time that hearing protection was used, and descriptions of exposure activities. It did not include questions on typical duration of each exposure, frequency of occurrence, or estimated sound level, and it is not clear how participants were to report multiple exposure activities. Finally, it is not clear how the data were combined to decide allocation to the high- or low-risk group; if quantitative methods were used, they were not reported. In contrast, the LENS-Q measure of Bramhall et al. (2017) is quantitative and well defined, but is effectively a measure of firearm exposure, discounting other forms of noise. Duration of each exposure is not considered, so a rifle round with a peak level of 160 dB SPL is equated to a long-duration exposure at 160 dB SPL. Put another way, one such rifle round is equated to one million heavy metal concerts (with a level of 100 dB SPL). This relative weighting is not supported by damage risk criteria (Nakashima, 2015).

The NESI aims to provide a more comprehensive measure of noise exposure, though administration can be time-consuming (5–35 min in the present study, depending on the extent and complexity of the respondent's noise history). Information is sought on all noisy activities experienced by the respondent, regardless of whether they are commonplace or unconventional and whether they occurred in occupational or recreational settings. For each activity, exposure habits may be expected to change across the lifespan. Hence, the NESI adopts a flexible, mnemonic approach, examining various life periods in which exposure habits were relatively stable. For each life period, rigorous methods are then applied in the estimation of sound level, duration, and usage and attenuation of hearing protection. Ultimately, a clearly defined method, based on the equal energy hypothesis, is used to combine the resulting data. Despite these properties, the NESI necessarily remains an inaccurate metric and it may therefore be important that our participants presented an extremely wide range of noise exposures, such that genuine differences were unlikely to be obscured by measurement error. Confidence in this interpretation – and in the measure – is bolstered by a previously reported association between tinnitus and noise exposure, as quantified using the NESI (Guest et al., 2017a).

ABR wave I amplitude may also be subject to doubt as a measure of synaptopathy in humans, despite clear correlations with synaptic loss in animal models (e.g., Kujawa and Liberman, 2009). In humans, the measure is contaminated by many non-synaptopathic sources of variability (Mitchell et al., 1989) and it has been suggested that within-subject difference measures might be necessary to emphasize AN function (Plack et al., 2016). However, such reasoning seems unlikely to account for the present results. In participants with SPiN difficulties, neither wave I amplitude nor wave I/V amplitude ratio was significantly reduced, despite small standard errors. Moreover, a trend was observed for *higher* wave I amplitude in those with SPiN difficulties (0.31 ± 0.03 μV) than in controls (0.27 ± 0.02 μV). A more fundamental defect of the ABR measures may be that wave I amplitude is not, after all, sensitive to loss of low-SR fibers. Bourien et al. (2014) demonstrated in the gerbil that fibers with the lowest SRs do not contribute to the compound action potential (equivalent to ABR wave I). A reasonable question is whether previously reported associations between wave I amplitude and noise exposure in humans (Bramhall et al., 2017; Stamper and Johnson, 2015b) reflect factors other than synaptopathy. Uncontrolled high-frequency or EHF audiometric loss may play a role (Guest et al., 2017b), since wave I is dominated by basal contributions (Don and Eggermont, 1978; Hardy et al., 2017), increasingly so at high stimulus levels (Eggermont and Don, 1980).

The EFR is thought to receive more robust contributions from low-SR fibers (Shaheen et al., 2015) and has some validation in animal models (Parthasarathy et al., 2017; Shaheen et al., 2015). Like the ABR, the EFR can be implemented using within-subject difference measures, in order to limit variability from non-synaptopathic factors. The present study used the variable-modulation-depth paradigm of Bharadwaj et al. (2015), which seeks to emphasize contributions of high-threshold fibers. Presence of SPiN difficulties was not associated with more steeply declining response strength, nor with reduced response strength overall. However, it is possible that our EFR stimuli – in common with those of other studies in humans – were inappropriate for the detection of synaptopathy. In animals, stimulus modulation rates of ∼1 kHz are required to elicit substantial AN contributions and disclose synaptopathy (Parthasarathy et al., 2017; Shaheen et al., 2015). Use of such high rates in humans presents significant challenges, potentially limiting the utility of the EFR as a measure of synaptopathy.

## 5 Conclusion

In individuals with impaired SPiN and normal audiograms, we find no evidence of enhanced lifetime noise exposure, nor of reduced brainstem response amplitudes. These results persist regardless of whether SPiN impairment is defined solely by self-report or confirmed by laboratory measures of SPiN. It is possible that the ABR and EFR measures offer limited sensitivity to cochlear synaptopathy, perhaps due to measurement variability from other sources or to limited contributions from low-SR AN fibres. Likewise, it is possible that the self-report measure of noise exposure lacks validity, despite its comprehensive nature and a previously reported association with tinnitus. Nevertheless, the resoundingly and uniformly null findings frustrate the notion that noise-induced cochlear synaptopathy is a significant etiology of SPiN impairment with a normal audiogram. It may be that synaptopathy alone does not have significant perceptual consequences, or is not widespread in humans with normal audiograms.

## Appendix A Supplementary data

Data Profile Data Profile
