# Supplementary material for: Impaired speech perception in noise with a normal audiogram: No evidence for cochlear synaptopathy and no relation to lifetime noise exposure
Source: Hear Res. 2018 Jul;364:142–51. doi: 10.1016/j.heares.2018.03.008 (PMC5993872; doi:10.1016/j.heares.2018.03.008)

Supplementary Analysis 1:

Reported-SPiN-impairment participants vs closely matched controls (n = 32 per group)

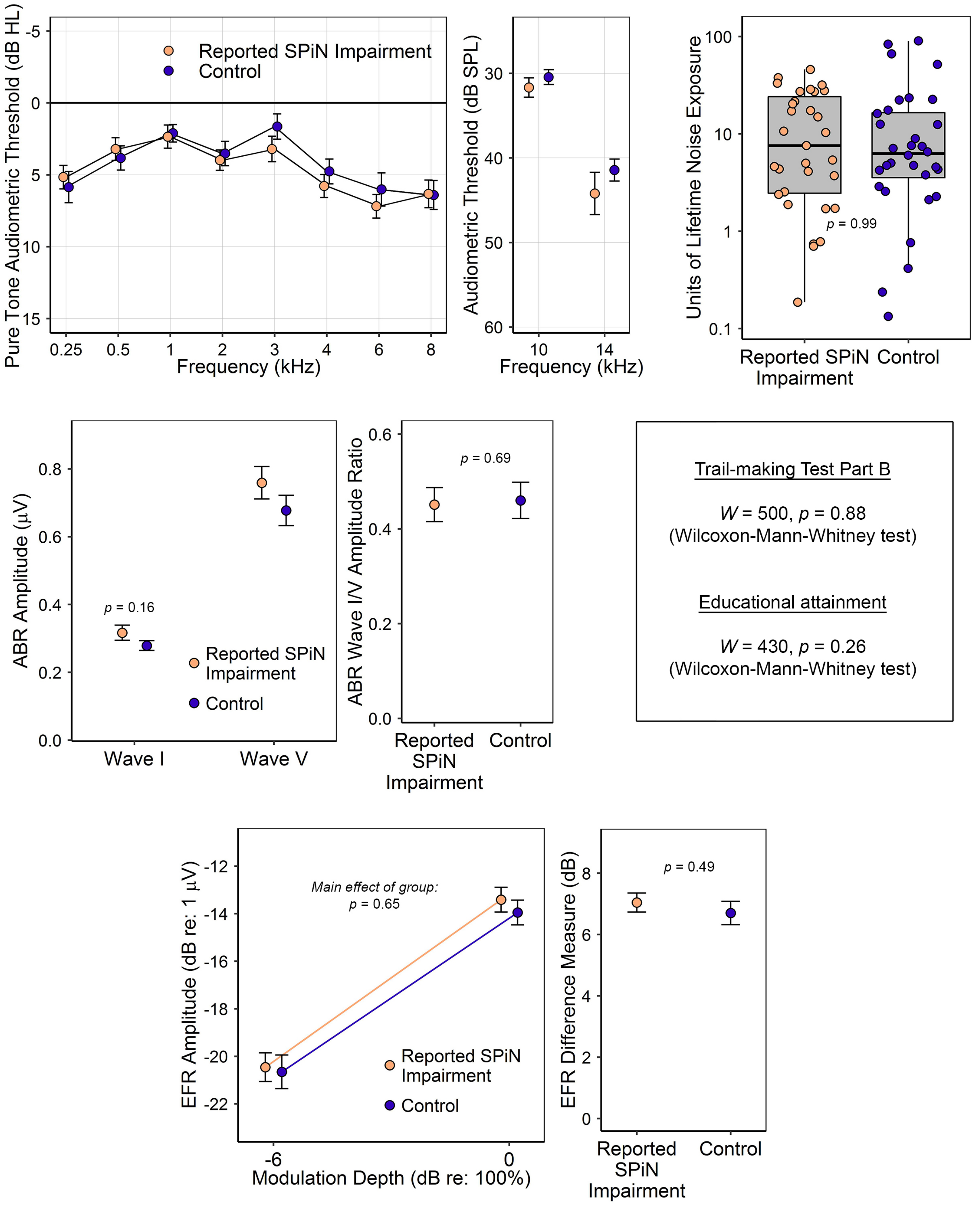

Supplementary Analysis 2:

Verified-SPiN-impairment participants vs non-audiogram-matched controls (n = 16 per group)

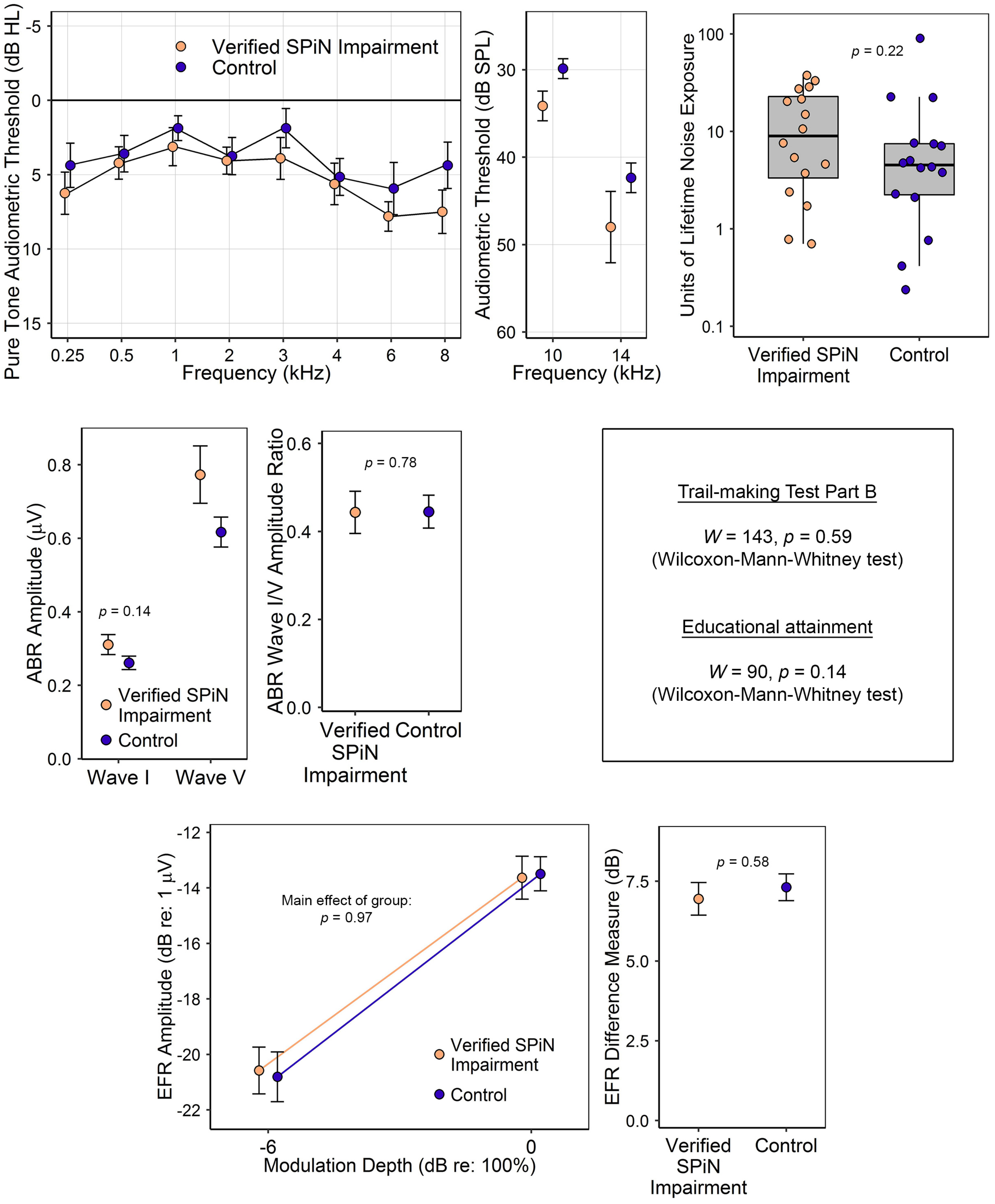

Table SM1: Characteristics of SPiN-impaired participants						
Age (years)	***Sex***	***CRM threshold (dB)***	***Verified SPiN impair-ment?***	***Description of speech perception difficulties***	***Time since onset***	***Any suspected cause?***
19.3	F	-9.1	Yes	Difficulties in BGN, despite having lots of experience listening in BGN (as a musician).	2 years (gradual)	Noise exposure (bar work)
20.2	F	-17.5	No	Difficulties anywhere with many competing voices, especially if music too (clubs & bars).	2 years (very gradual)	Noise exposure (playing in orchestra)
21	F	-18.1	No	Has to ask people to repeat themselves if multiple talkers (e.g. at church social events).	Not sure	No
23.5	F	-10.5	Yes	Difficulties if many voices at once, even if no music (e.g. pub). Also when in traffic noise.	Several years (very gradual)	Noise exposure
24.8	F	-9.1	Yes	Difficulties following speech, especially if there are voices or music in the background.	10 years (gradual)	Loud earphone use
25.3	F	-11.9	Yes	Great difficulty focussing in BGN, especially if multiple talkers involved in the conversation. Relies on lipreading. Happens in restaurants, not just in higher noise levels.	Many years, but deteriorated over past few	Perhaps hereditary (father has hearing loss)
25.3	F	-15.4	No	Difficulties in BGN, but sometimes even in quiet.	3 years (gradual)	No
25.6	F	-13.5	Yes	Difficulties following conversation in nightclubs and noisy bars. Relies on lipreading.	2 years (gradual)	Noise exposure
27.9	F	-16.3	No	Partner says that she mishears a lot. Has to rely on lipreading in bars and clubs.	3 years (gradual)	Noise exposure (nightclubs)
28	F	-15.4	No	Difficulties if more than one person talking, even if not very loud (e.g. staff room at work).	A few years (gradual)	No
29.5	F	-19.9	No	Difficulties following speech in BGN or if talker's face not visible.	1 year (gradual)	Noise exposure as dental nurse (drilling, suction)
29.9	F	-9.3	Yes	Difficulties in noisy restaurants and bars.	<17 years (very gradual)	No
30.2	F	-16.0	No	Difficulties if multiple talkers, even if not loud. Has asked her friends to adapt their communication with her to help manage her listening difficulties.	5 years (gradual)	Perhaps concerts, but hasn't been to many
31.3	F	-12.1	Yes	Great difficulties in BGN, whether music or multiple voices. Tends to tune out mentally after a while. Also can't hear her own voice, causing her to shout.	14-19 years	No
32.4	F	-10.5	Yes	Difficulties if lots of people talking, or if (e.g.) TV on in background.	Several years (gradual)	Noise exposure (especially nightclubs)
33.9	F	-10.3	Yes	Difficulties following conversation in BGN, even just in a café.	3 years (gradual)	Noise exposure
35.4	F	-16.5	No	Difficulties if multiple talkers, BGN, reverberation, or if talking on speakerphone.	A long time, but greatly deteriorated in past year	Loud music listening when younger
37.8	F	-11.1	Yes	Difficulties in BGN.	10 years (gradual)	Ageing & mild noise damage
18.8	M	-15.0	No	Difficulties in restaurants and bars. Also in constant BGN (e.g. extractor fan).	2 years (gradual)	No
19.7	M	-17.5	No	Difficulties understanding speech in bars, even if not very loud and just multiple talkers.	At least 2 years	No
19.7	M	-13.6	Yes	Difficulties in bars and clubs, but also difficulties when not very loud: background music or engine noise.	8-9 years, but deteriorated over past 4	Possibly hereditary
20.5	M	-14.5	No	Difficulties in BGN, whether made up of loud voices or music. Needs subtitles for some TV.	Not sure, perhaps very long-term	No
21.1	M	-5.5	Yes	Loss of auditory clarity. Mishears if any competing talkers.	3 years (very gradual)	Noise exposure (gigs, DJing)

21.3	M	-11.4	Yes	Difficulties in BGN, whether loud music or just multiple talkers.	3 years (gradual)	Exposure to loud music
21.4	M	-15.5	No	Difficulties with auditory selective attention when multiple sound sources. Parents noticed difficulties.	7-8 years	No
22.3	M	-15.3	No	Has to ask people to repeat themselves, sometimes three or four times. Difficulties if multiple talkers, even if not very loud.	2 years (gradual)	Loud music (earphones & club nights)
24.1	M	-14.1	No	ENT diagnosed with suspected APD.	6-7 years (gradual)	No
30	M	-21.4	No	Difficulties in BGN, e.g. competing voices or bus noise. Mishears target voice.	6 years (gradual)	No
30.8	M	0.4	Yes	Difficulties in BGN, particularly understanding certain voices. Embarrassing. Worse in high pressure situations.	11-13 years (very gradual)	Combination of hearing damage & stress
31.9	M	-12.3	Yes	In childhood, struggled to hear instructions in noisy classrooms. Parents were aware. Now has difficulties anywhere with lots of voices, with or without music (e.g. restaurants).	~20 years	No
33.6	M	-8.3	Yes	Difficulties in BGN.	2-3 years (fairly rapid decline, but no known cause)	No
34.5	M	-16.5	No	Difficulties in any situation with significant BGN (doesn't have to be loud – any complex auditory environment).	10-14 years (gradual)	No

**Table SM2: Conversion of noise exposure levels from participant estimate to dBA**			
Free-field exposures		***Personal listening devices***	
Required vocal effort	***Estimated level***	***Volume control setting***	***Estimated level***
Normal voice at 1.2m	**< 80 dBA**	<70% of maximum	**< 80 dBA**
Raised voice at 1.2m	**87 dBA**	70% of maximum	**82 dBA**
Loud voice at 1.2m	**90 dBA**	80% of maximum	**88 dBA**
Very loud voice at 1.2m	**93 dBA**	90% of maximum	**94 dBA**
Shouting at 1.2m	**99 dBA**	Maximum volume	**100 dBA**
Shouting at 0.6m	**105 dBA**		
Shouting at listener's ear	**110 dBA**		

Table SM3: Noise exposure calculation for a single participant							
Exposure activity	Bar work in nightclub	Festivals (acoustic)	Gigs (at 18 to 31)	Gigs (at 31 to 36)	Nightclubs (at 16 to 22)	Nightclubs (at 22 to 31)	Nightclubs (at 31 to 36)
Additional information	Music on throughout shift	15 one-day festivals (all in past 5 years)	~8 per year	~2 per year	~2 nights per week during term time	~4 nights per week, 40 weeks per year	Once every ~3 months
Sound level descriptor	Shout at 0.6m	Talk very loudly at 1.2m	Shout at 0.6m	Shout at 0.6m	Shout at 1.2m	Shout at 1.2m	Shout at 1.2m
Estimated level (dBA)	**105**	**93**	**105**	**105**	**99**	**99**	**99**
Years	1	5	13	5	6	9	5
Weeks/year	52	3	8	2	40	40	4
Days/week	3	1	1	1	2	4	1
Hours/day	4	12	3	3	5	5	5
Total duration (hrs)	**624**	**180**	**312**	**30**	**2400**	**7200**	**100**
Type of hearing protector	None	3M foam plugs	None	None	None	None	None
Attenuation (dB)		21					
Proportion of time worn		10%					
Exposure units	**9.49**	**0.16**	**4.74**	**0.46**	**9.17**	**27.50**	**0.38**
TOTAL UNITS OF LIFETIME NOISE EXPOSURE = 51.89							

		Table SM4: Electrophysiological results				
		ABR wave I amplitude (µV)	***ABR wave I/V amplitude ratio***	***EFR amplitude (dB re: 1 µV)***		***EFR difference measure (dB)***
				-6 dB depth	*0 dB depth*	
Main analysis	*Verified SPiN impairment*	**0.311** ± 0.027	**0.443** ± 0.048	**-20.58** ± 0.84	**-13.63** ± 0.77	**6.95** ± 0.51
	Audiogram-matched controls	**0.288** ± 0.017	**0.447** ± 0.039	**-20.52** ± 1.00	**-13.65** ± 0.75	**6.87** ± 0.56
Supplementary analysis 1	*Reported SPiN impairment*	**0.317** ± 0.022	**0.451** ± 0.036	**-20.46** ± 0.60	**-13.42** ± 0.52	**7.05** ± 0.31
	Audiogram-matched controls	**0.279** ± 0.014	**0.460** ± 0.038	**-20.65** ± 0.71	**-13.95** ± 0.52	**6.70** ± 0.38
Supplementary analysis 2	*Verified SPiN impairment*	**0.311** ± 0.027	**0.443** ± 0.48	**-20.58** ± 0.84	**-13.63** ± 0.77	**6.95** ± 0.51
	Non-audiogram-matched controls	**0.261** ± 0.018	**0.445** ± 0.037	**-20.81** ± 0.90	**-13.49** ± 0.61	**7.31** ± 0.42

Electrophysiological recording and analysis methods

Participants reclined with eyes closed in a double-walled, sound-attenuating booth. Auditory stimuli were presented via electromagnetically shielded ER3A insert earphones driven by an Avid FastTrack C400 audio interface. A BioSemi Active2 measurement system recorded from active electrodes at Cz, C7, and both mastoids. Common Mode Sense and Driven Right Leg electrodes were attached at mid-forehead and electrode offsets remained within ±40 mV throughout all recordings. Data streams from all four electrodes were saved for offline analysis, along with stimulus-timing information received from the external audio interface via a custom-made trigger box.

Auditory brainstem response

Stimuli were designed to focus excitation on the characteristic frequencies typically affected by early noise-induced cochlear damage. 100 µs pulses were high-pass filtered (first-order butterworth, 2.4 kHz cutoff) and delivered via ER3A inserts, yielding clicks whose 10 dB bandwidth extended from 1.2 to 4.7 kHz (as recorded in a Gras IEC60711 occluded-ear simulator). The stimuli were delivered at a level of 102 dB peSPL, sufficient to elicit the half-octave basalward shift in the travelling wave (McFadden, 1986) and provide strong excitation of characteristic frequencies between approximately 2 and 7 kHz. Each ear received 7040 clicks at a rate of 7.05/second. However, presentation alternated between ears, leading to an overall presentation rate of 14.1/second and halving the recording time. The inter-stimulus interval was jittered by up to 10%, in order to prevent the accumulation of stationary interference.

Bioelectrical activity between Cz and ipsilateral mastoid was recorded at a sampling rate of 16384 Hz and divided into epochs extending from 10 ms pre-stimulus to 8 ms post-stimulus. Epochs whose activity exceeded the mean for the recording by more than two standard deviations were rejected. Those that remained were averaged and the resulting waveforms were filtered between 50 and 1500 Hz (fourth-order butterworth) and corrected for any linear drift by subtracting a linear fit to the pre-stimulus baseline. Waves I and V were then quantified by a peak-picking algorithm that identified features in specified time windows. Wave I was defined as a maximum occurring 1.55-2.05 ms after stimulus peak; wave V as a maximum (or inflection point on a falling portion of the waveform) occurring 5.1-6.6 ms after stimulus peak; the trough of wave I as the lowest point occurring 0.3-1.0 ms after the peak of wave I. Wave I amplitude was measured from peak to trough; wave V amplitude from peak to pre-stimulus baseline. Post-hoc subjective review verified that the algorithm had appropriately interpreted all waveforms (presented in full on pages 7 and 8 of the supplementary material).

Envelope-following response

Stimuli were 75 dB SPL transposed tones (Bernstein and Trahiotis, 2002) with carrier frequency 4000 Hz and modulation rate 100 Hz. In order to attenuate off-frequency contributions, tones were presented concurrently with a notched-noise masker (bandwidth 20-10000 Hz, notch width 800 Hz), realized separately for each trial and applied at an SNR of 20 dB (broadband RMS). Stimulus duration was 400 ms with the addition of 15 ms onset and offset ramps. The duration of the inter-stimulus interval was 400 ms on average, jittered by up to 10%. Following the methods of Bharadwaj et al. (2015), tones were of two modulation depths: 0 dB (full modulation) and -6 dB (shallow modulation). Each of these tones was presented 1260 times, half in each polarity. The resulting four stimuli were interleaved throughout the recording, in the sequence 0 dB; 0 dB inverted; -6 dB; -6 dB inverted.

Bioelectrical activity between Cz and C7 was extracted for epochs extending from 4 to 404 ms after the end of the stimulus onset ramp. For each stimulus modulation depth and polarity, epochs were rejected if their RMS activity exceeded the 99th percentile for recording. The remaining epochs were averaged and the responses to opposing polarities summed, emphasizing the response to the temporal envelope. Each resulting EFR was subjected to a discrete Fourier transform to yield the response amplitude (at the 100 Hz modulation frequency) and an estimate of the noise floor (based on activity in 10 adjacent frequency bins).

Following Bharadwaj et al. (2015), we aimed to enhance sensitivity to cochlear synaptopathy by computing an EFR difference measure: the difference in response amplitude (in dB) at the two stimulus modulation depths. This measure is closely related to the “EFR slope” metric of Bharadwaj and colleagues, though based on a two-point rather than a three-point function. Such measures rest on the assumption that synaptopathy preferentially affects high-threshold AN fibers and should therefore preferentially degrade the encoding of stimuli with shallow modulations. A schematic illustration of the difference measure is provided in Fig. 1. Since it is possible that responses to both modulation depths might be impaired by synaptopathy, raw response amplitude was also analyzed.

Peak-picked ABRs (SPiN-impaired participants)

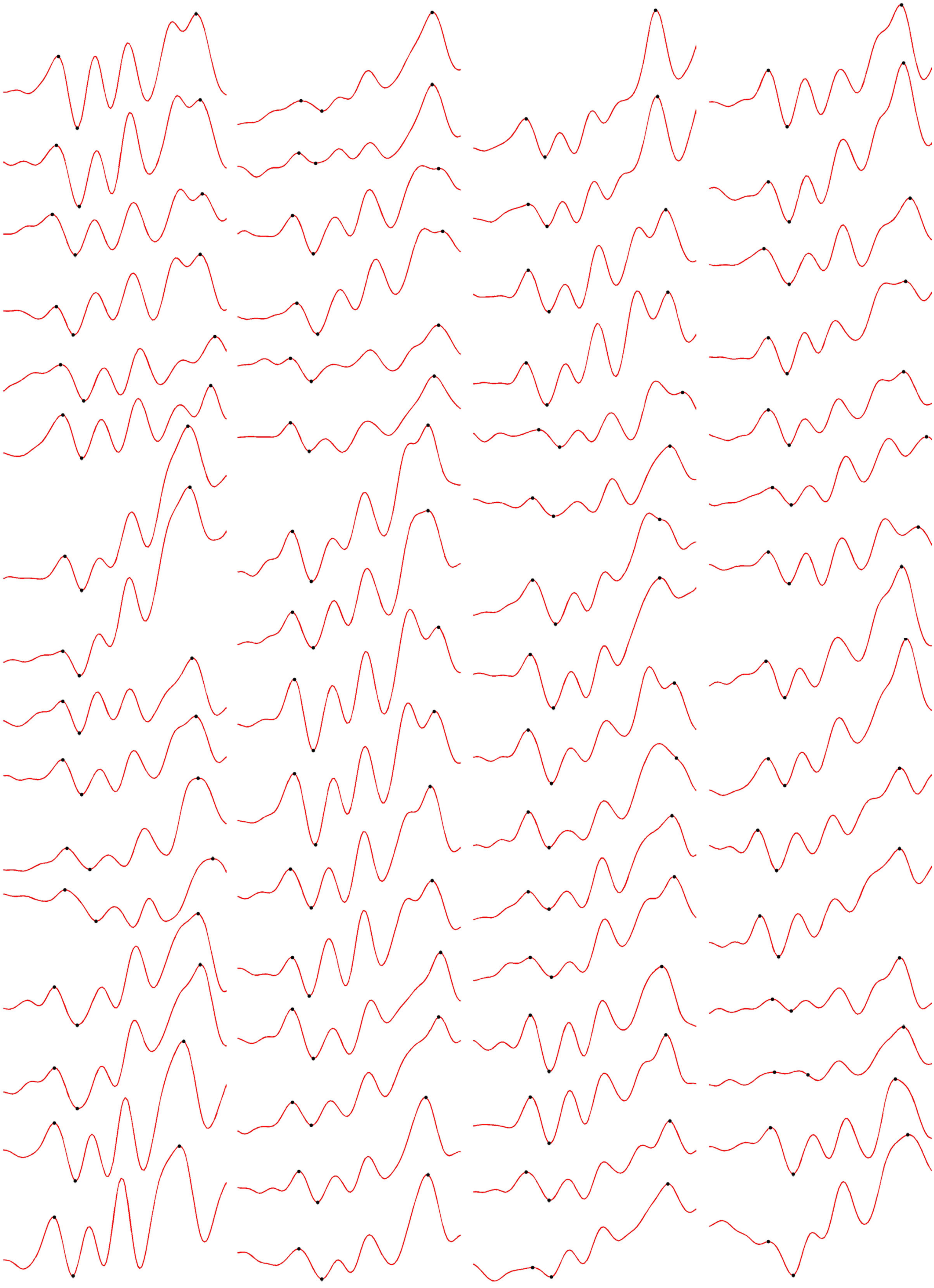

Peak-picked ABRs (control participants)

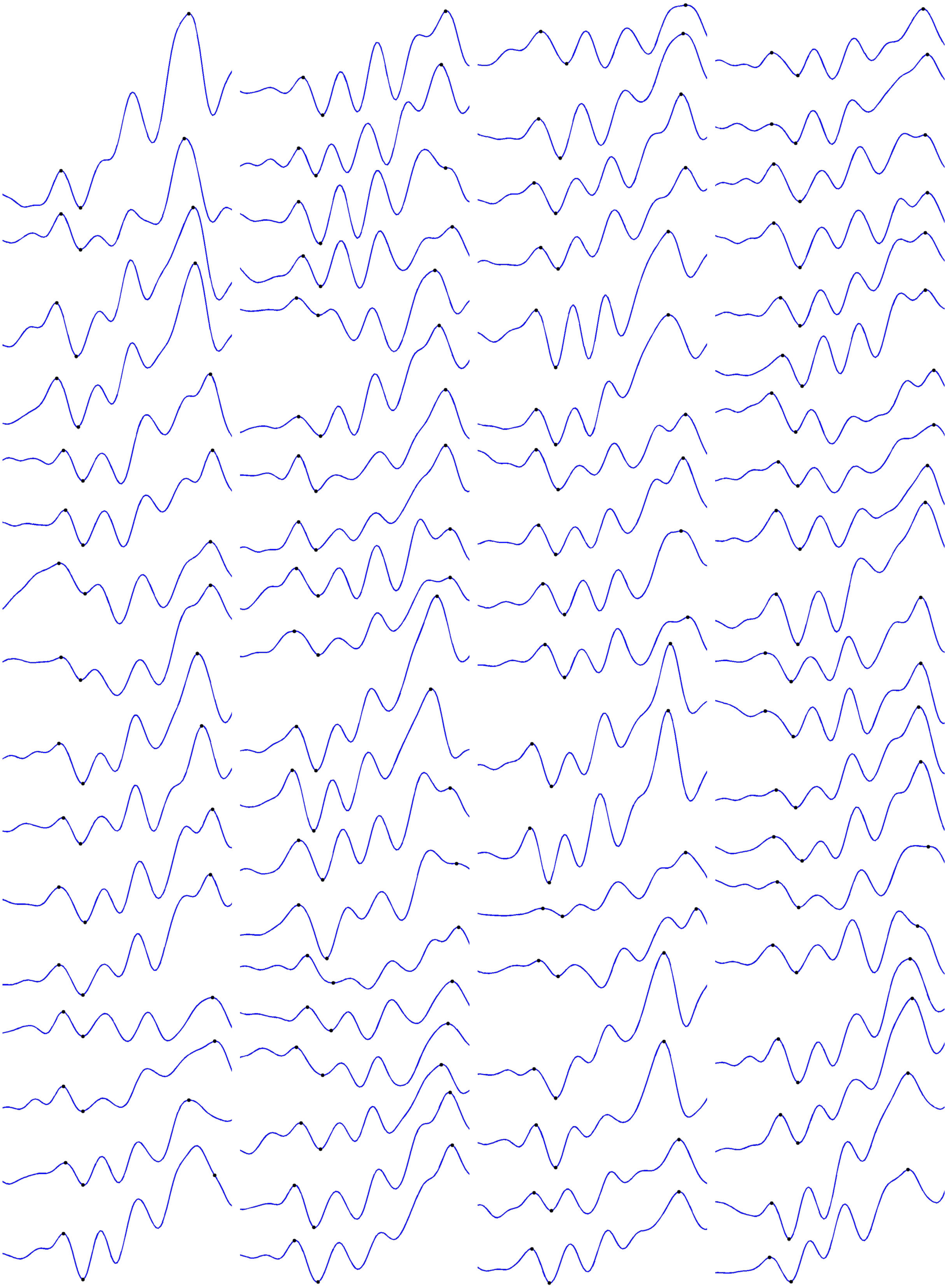

Supplement: Supplementary material [file mmc2.pdf]
